# Generalized Morphea Successfully Treated With Tripterygium Glycosides and Hydroxychloroquine: A Case Report and a Systematic Review

**DOI:** 10.7759/cureus.102597

**Published:** 2026-01-29

**Authors:** Zufeng Sun, Jingjing Chen, Xiulian Xu, Jiahui Zhang, Xu Yao

**Affiliations:** 1 Department of Allergy and Rheumatology, Hospital for Skin Diseases, Institute of Dermatology, Chinese Academy of Medical Sciences &amp; Peking Union Medical College, Nanjing, CHN; 2 Department of Pathology, Hospital for Skin Diseases, Institute of Dermatology, Chinese Academy of Medical Sciences &amp; Peking Union Medical College, Nanjing, CHN; 3 Department of Sexually Transmitted Disease Epidemiology, Hospital for Skin Diseases, Institute of Dermatology, Chinese Academy of Medical Sciences &amp; Peking Union Medical College, Nanjing, CHN

**Keywords:** generalized morphea, glucocorticoid, hydroxychloroquine, treatment, tripterygium glycosides

## Abstract

Generalized morphea, a rare and severe variant of localized scleroderma, is marked by extensive skin involvement with multiple hardened plaques and hyperpigmentation. The pathogenesis of generalized morphea is still unclear, and there is no established therapy. The aim of this article is to present a novel treatment approach for generalized morphea while also conducting a systematic review of previously published cases to summarize and analyze the clinical features and current effective treatment strategies for this condition. We herein describe the case of a 61-year-old male patient with generalized morphea successfully treated with tripterygium glycosides (TG) and hydroxychloroquine (HCQ). The systematic review analyzed a total of 77 patients with generalized morphea, comprising 76 previously reported cases (57 adults and 19 children) and one adult patient from our article. Compared with the adult group, the pediatric group demonstrated significantly higher rates of abnormal antinuclear antibodies (78.9%, p=0.002) and a greater proportion of severe cases (89.5%, p=0.001). Furthermore, systemic glucocorticoid is currently the most frequently used and effective treatment (p=0.016). In the treatment of generalized morphea, the combination of HCQ and TG may represent a potentially useful therapeutic option worthy of further investigation.

## Introduction

Morphea, also known as localized scleroderma, is a rare disease of unknown etiology and characterized by skin induration and sclerosis, with increased quantities of collagen in the lesion. It includes five clinical subtypes: circumscribed, linear, generalized, pansclerotic, and mixed [1]. Generalized morphea is a form of localized scleroderma characterized by four or more plaques exceeding 3cm in size, which may merge and involve at least two different body regions [2]. The plaques are usually present on the trunk and extremities. In severe cases, patients may present with symptoms such as skin ulcers, restricted joint mobility, and joint pain, which can significantly impact quality of life and be life-threatening. Although the specific pathogenesis of generalized morphea is unknown, several factors are recognized as triggers, including infection, drugs, radiation exposure, and mechanical factors such as physical exercise, insect bite, and accidental trauma [3,4]. As a rare skin disorder, it currently lacks randomized controlled studies or well-designed clinical trials for treatment. Consequently, existing management approaches are primarily derived from case reports. We present the first case of generalized morphea successfully treated with a combination of tripterygium glycosides (TG) and HCQ. Furthermore, a systematic literature review was performed to investigate the clinical features and evaluate effective therapeutic options.

## Case presentation

A 61-year-old male patient presented with a three-month history of sclerotic skin and erythema on the trunk and limbs. Three months ago, following an insect bite, the patient began to develop scattered erythematous and sclerotic plaques on the trunk, accompanied by mild itching. Subsequently, similar skin lesions gradually spread to the limbs and partially coalesced into larger patches. During the course of the disease, the patient experienced mild pruritus with no other symptoms. He had a one-year history of hypertension. There was no history of infection, drug use, or toxic exposure.

**Figure 1:**
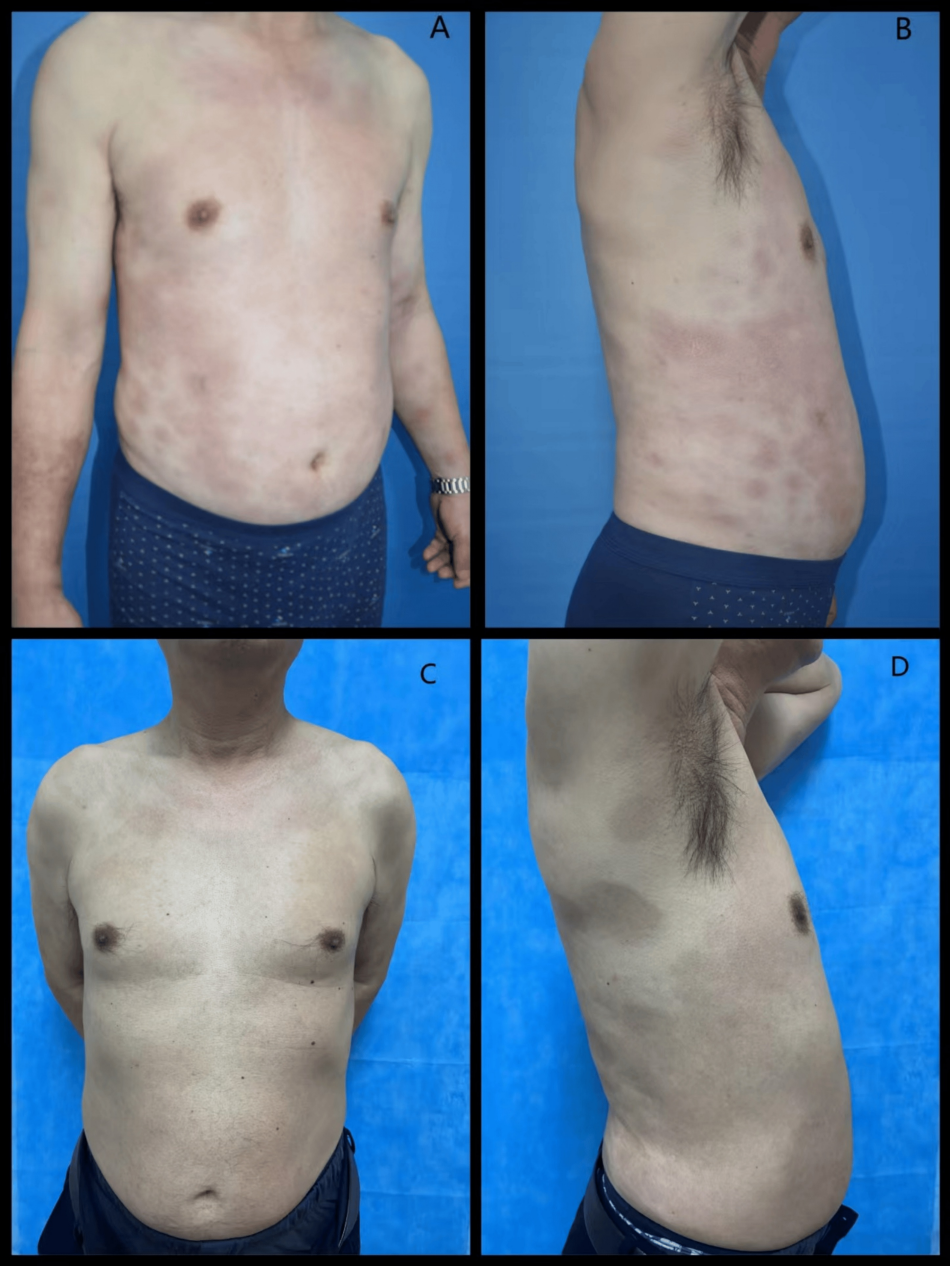
Treatment effect of TG and HCQ on the patient with generalized morphea. (A and B) Multiple indurated erythematous plaques on the trunk and limbs before treatment. (C and D) Residual hyperpigmented patches on the trunk after treatment. TG, tripterygium glycosides; HCQ, hydroxychloroquine

Physical examination revealed multiple indurated erythematous plaques on the trunk and limbs (Figures 1A, 1B).

**Figure 2:**
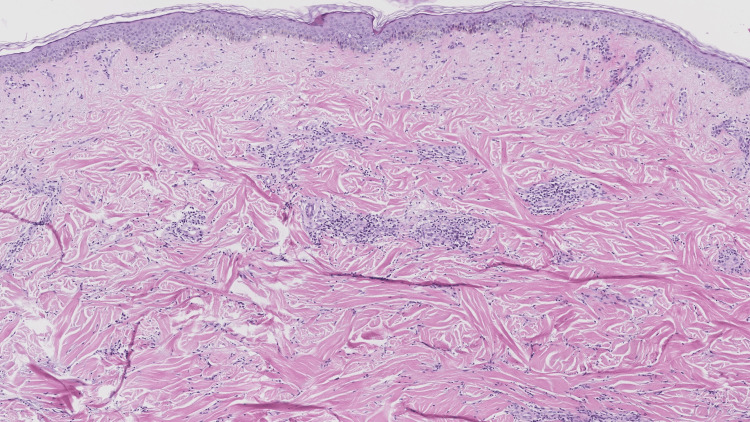
Histopathological result of the biopsy from the lesion of the patient with generalized morphea. Proliferated collagen fibers, thickened collagen bundles, and focal lymphocyte infiltrates in the dermis can be seen (H&E, ×50).

No lesions were presented on the face, hands, or feet. Laboratory tests showed a marginally elevated eosinophil count (8.5%, normal value: 0.5-5%). Autoimmune markers, C-reactive protein levels, and liver and renal function were all within normal limits. A skin biopsy taken from the abdomen revealed proliferated collagen fibers, thickened collagen bundles, and focal lymphocyte infiltrates in the dermis (Figure 2). Based on clinical and histopathological findings, generalized morphea was diagnosed. Based on the absence of sclerodactyly, visceral involvement, Raynaud's phenomenon, and negative specific autoantibodies (anti-Scl-70/ACA), the condition was distinguishable from systemic sclerosis. The patient was treated with TG (60 mg daily) and HCQ (0.4 g daily). After three months of therapy, the skin lesions showed remarkable improvement. The patient subsequently discontinued all pharmacological treatment. At the four-year follow-up, the skin lesions had completely resolved, with only residual pigmentation remaining (Figures 1C, 1D). During the treatment period, monitoring of the complete blood count and liver/kidney function showed normal results, and no ocular discomfort symptoms occurred.

## Discussion

Literature review

Methods

We performed a literature search in PubMed, Embase, and Ovid databases employing the search terms ("generalized morphea" OR "generalized localized scleroderma") AND "treatment" for the period of 1990 to 2025, limited to human case reports in English. For inclusion in this systematic review, studies had to meet the following criteria: (1) a definitive diagnosis of generalized morphea and (2) well-documented clinical data, including detailed treatment regimens and clearly reported therapeutic outcomes.

**Figure 3:**
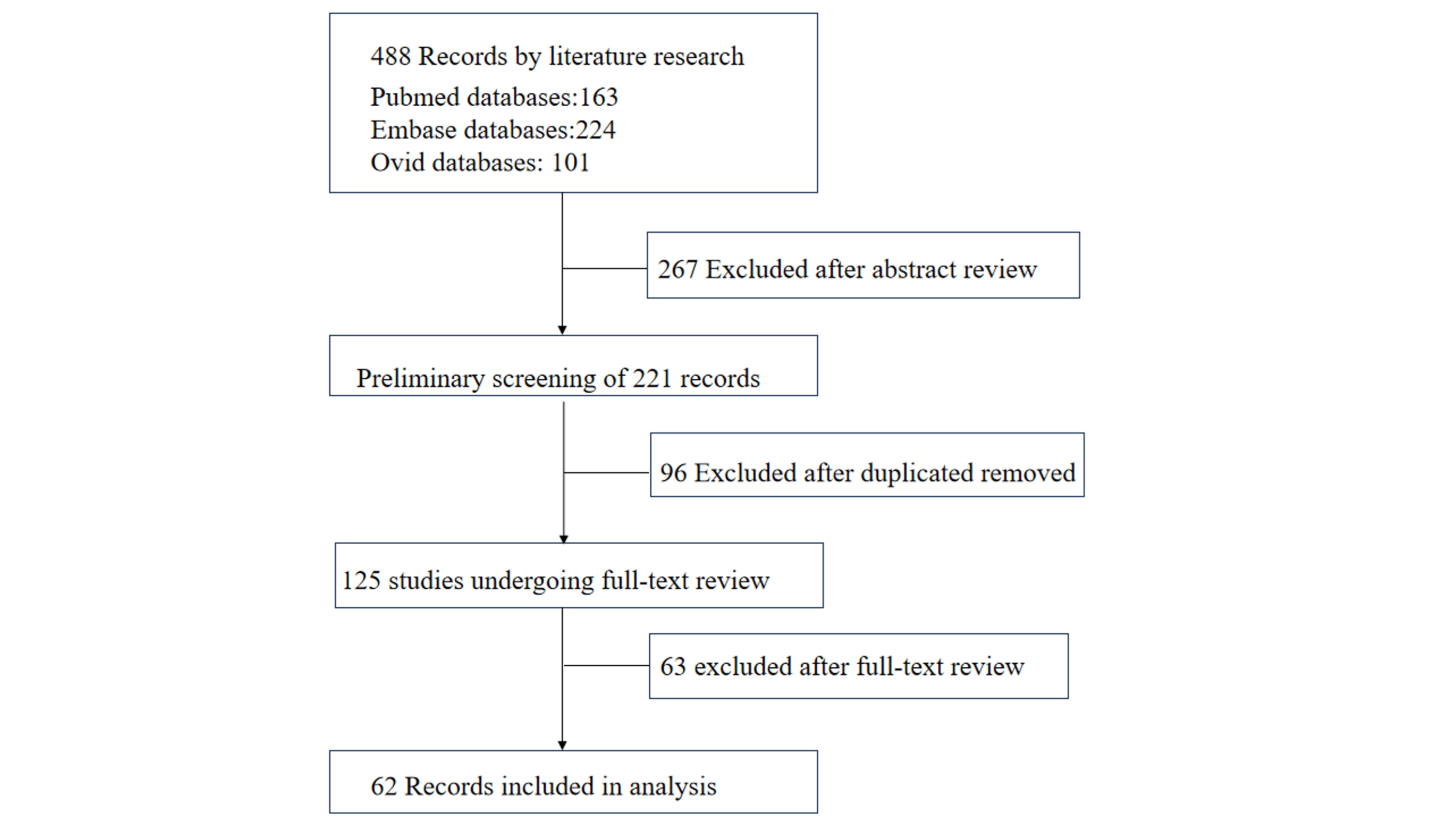
Flowchart of literature search and study selection

Of the 488 reports generated, 62 met the inclusion criteria (Figure 3), comprising 19 pediatric and 57 adult cases. A severe case was identified as any patient presenting with skin sclerosis accompanied by symptoms such as skin ulcers, limb or face atrophy, decreased range of motion over joint, joint pain, or leg length discrepancy. Treatment outcomes were categorized as effective or ineffective according to predefined criteria. An effective treatment response was defined as the softening, reduction, or complete resolution of sclerotic plaques, accompanied by improved range of motion and reduced pain in affected joints. Conversely, ineffective treatment referred to either the absence of measurable clinical improvement or the evidence of disease progression following therapy.

Statistical analyses were performed using SPSS software version 23.0 (IBM Corp., Armonk, NY). Data are expressed as the means ± SD or as a percentage. The t-test, chi-square test (χ2) test, and Fisher’s exact test were used to compare proportions between groups. A p-value of <0.05 was considered statistically significant.

Results

This study comprised a total of 77 patients, including 19 pediatric cases and 58 adult cases. The mean age of children was 8.4±5.0 years, while the mean age of adults was 56.4±14.6 years. Among all patients, 37 had abnormal antinuclear antibodies, and the proportion of abnormal antinuclear antibodies was significantly higher in the pediatric group (78.9%) compared to the adult group (37.9%) (p=0.002). As shown in Table 1, no significant differences were observed between the two groups in the proportion of patients with elevated eosinophil, ESR, or CRP levels. Among adult patients with generalized morphea, the most common comorbidity with autoimmune diseases was eosinophilic fasciitis (seven cases, 12.1%), followed by primary biliary cirrhosis (four cases, 6.9%). Severe cases were significantly more frequent in the pediatric group (17 cases, 89.5%) than in the adult group (26 cases, 44.8%) (p=0.001).

**Table 1:** Clinical characteristics of patients with generalized morphea. *Denotes the t-value. CRP; C-reactive protein; ESR, erythrocyte sedimentation rate

Characteristic	Pediatric Group (n=19)	Adult Group (n=58)	t/χ²	p-Value
Demographics				
Age, years, mean ± SD	8.4 ± 5.0	56.4 ± 14.6	–	–
Female, n (%)	16 (84.2)	37 (63.8)	2.781	0.095
Disease duration, months, mean ± SE	45.7 ± 9.5	19.5 ± 5.8	1.549*	0.126
Laboratory abnormalities, n (%)				
Positive antinuclear antibodies (normal range ≤1:40)	15 (78.9)	22 (37.9)	9.645	0.002
Elevated eosinophils (normal range: 0.05-0.5×10^9^/L)	4 (21.1)	16 (27.6)	-	0.765
Elevated CRP/ESR (CRP normal range: 0-10 mg/L; ESR normal range: 0-20 mm/h)	4 (21.1)	11 (19.0)	-	1.000
Comorbid autoimmune disease, n (%)				
Systemic sclerosis	0 (0.0)	1 (1.7)	-	1.000
Necrotizing vasculitis	1 (5.3)	0 (0.0)	-	0.247
Primary biliary cholangitis	0 (0.0)	4 (6.9)	-	0.567
Eosinophilic fasciitis	0 (0.0)	7 (12.1)	-	0.183
Sjögren's syndrome	0 (0.0)	1 (1.7)	-	1.000
Polymyositis	1 (5.3)	1 (1.7)	-	0.435
Disease severity, n (%)				
Severe cases	17 (89.5)	26 (44.8)	11.569	0.001

We systematically recorded all therapeutic regimens administered (either as monotherapy or combination therapy, and classified as effective or ineffective) to fully assess treatment outcomes. Since patients often received multiple sequential regimens (e.g., an ineffective initial therapy followed by an effective or ineffective subsequent ones), the total percentage exceeded 100%. In total, 96 therapeutic regimens were recorded, of which 25 were ineffective and 71 were effective. Our study found that systemic glucocorticoid, methotrexate, and HCQ were the most commonly used therapeutic options at present. Statistical analysis revealed that glucocorticoid (65.6%, p=0.016) was associated with a significantly higher proportion of effective cases, whereas HCQ was associated with significantly more ineffective cases (75%, p=0.004). On intergroup comparison, no statistically significant differences in effectiveness were observed for ciclosporin, azathioprine, cyclophosphamide, mycophenolate mofetil, intravenous immunoglobulin, calcitriol, TNF-α inhibitors, Janus kinase inhibitors, and other agents (Table 2).

**Table 2:** Comparison of the efficacy of different drug therapies for generalized morphea. MTX, methotrexate; HCQ, hydroxychloroquine; MMF, mycophenolate mofetil; AZA, azathioprine; TNF-α, tumor necrosis factor-alpha; CTX, cyclophosphamide; IVIg, intravenous immunoglobulin; JAK, Janus kinase; UVA, ultraviolet A; PUVA, psoralen and ultraviolet A

	Effective (n = 71)	Ineffective (n = 25)	p-Value
Corticosteroids, n (%)	40 (65.6)	21 (34.3)	0.016
MTX, n (%)	19 (65.5)	10 (34.5)	0.231
HCQ, n (%)	2 (25)	6 (75)	0.004
Ciclosporin, n (%)	4 (66.7)	2 (33.3)	0.652
MMF, n (%)	4 (66.7)	2 (33.3)	0.652
AZA, n (%)	2 (40)	3 (60)	0.112
TNF-α, n (%)	4 (66.7)	2 (33.3)	0.652
CTX, n (%)	2 (50)	2 (50)	0.282
Calcitriol, n (%)	6 (100)	0	0.335
IVIg, n (%)	4 (100)	0	0.570
Imatinib, n (%)	4 (100)	0	0.570
Tetracycline, n (%)	4 (80)	1 (20)	1.000
JAK inhibitors (%)	3 (100)	0	0.564
UVA, n (%)	4 (57.1)	3 (42.9)	0.375
PUVA, n (%)	2 (40)	3 (60)	0.112

Furthermore, comparison between systemic glucocorticoid monotherapy and glucocorticoid-based combination therapy revealed no significant differences in effectiveness (χ²=0.135, p=0.713) (Table 3).

**Table 3:** Efficacy of systemic glucocorticoid monotherapy versus combination therapy in generalized morphea

Systemic glucocorticoid	Effective (n=37)	Ineffective (n=17)	χ^2^	p-Value
Monotherapy group	15	6	-	-
Combined therapy group	22	11	0.135	0.713

Our finding indicates that generalized morphea predominantly affects adults, while pediatric patients exhibit a higher rate of abnormal antinuclear antibody positivity and a greater proportion of severe cases. In addition to antinuclear antibodies, other immunological abnormalities including antibodies against single or double-stranded DNA, anti-Scl-70, anti-snRNP, and anti-Ku antibodies have also been observed in patients with generalized morphea [5,6]. Commonly associated symptoms include dysphagia, joint pain, and Raynaud phenomenon [4]. Our study also identified comorbidities such as eosinophilic fasciitis, primary biliary cholangitis, Sjögren's syndrome, and polymyositis. Taken together, these collective findings supported the proposition that generalized morphea represents a systemic autoimmune syndrome rather than a skin-limited disorder [5].

Although no treatment consensus exists for generalized morphea, our systematic review of published cases indicates that systemic glucocorticoid appears to be the most frequently used intervention and that it is associated with the highest rates of clinical improvement. Despite the general inefficacy of HCQ for generalized morphea identified by our statistical analysis, the patient described in our case report responded favorably. This clinical improvement might be attributed to the concurrently administered TG. TG possesses known anti-inflammatory and immunosuppressive properties [7,8] and is widely used in China for treating autoimmune diseases such as rheumatoid arthritis and systemic lupus erythematosus [9,10]. While other treatments, including IVIG, calcitriol, JAK inhibitors, and imatinib demonstrated a 100% response rate in the limited cases available, no statistically significant differences in efficacy could be established due to small sample sizes. Further clinical data are needed to validate these findings.

## Conclusions

In this article, we report a case of generalized morphea successfully treated with a combination of HCQ and TG. The combination of HCQ and TG is a potentially useful therapeutic option for generalized morphea that warrants further study. We also present the first systematic literature review on the clinical features and treatment of this disease. Systemic glucocorticoid is currently the most frequently used and effective treatment for generalized morphea. However, further research is needed to evaluate the efficacy of such regimens in generalized morphea.

## References

[1] Laxer RM, Zulian F (2006). Localized scleroderma. Curr Opin Rheumatol.

[2] Chung L, Lin J, Furst DE, Fiorentino D (2006). Systemic and localized scleroderma. Clin Dermatol.

[3] Careta MF, Romiti R (2015). Localized scleroderma: clinical spectrum and therapeutic update. An Bras Dermatol.

[4] Zulian F, Athreya BH, Laxer R (2006). Juvenile localized scleroderma: clinical and epidemiological features in 750 children. An international study. Rheumatology (Oxford).

[5] Leitenberger JJ, Cayce RL, Haley RW, Adams-Huet B, Bergstresser PR, Jacobe HT (2009). Distinct autoimmune syndromes in morphea: a review of 245 adult and pediatric cases. Arch Dermatol.

[6] Kishi T, Miyamae T, Morimoto R (2015). Childhood-onset anti-Ku antibody-positive generalized morphea with polymyositis: a Japanese case study. Pediatr Dermatol.

[7] Lin N, Zhang YQ, Jiang Q (2020). Clinical practice guideline for tripterygium glycosides/Tripterygium wilfordii tablets in the treatment of rheumatoid arthritis. Front Pharmacol.

[8] Hu T, Shi C, Liu L, Li P, Sun Y, An Z (2020). A single-injection targeted metabolomics profiling method for determination of biomarkers to reflect tripterygium glycosides efficacy and toxicity. Toxicol Appl Pharmacol.

[9] Wang HL, Jiang Q, Feng XH (2016). Tripterygium wilfordii Hook F versus conventional synthetic disease-modifying anti-rheumatic drugs as monotherapy for rheumatoid arthritis: a systematic review and network meta-analysis. BMC Complement Altern Med.

[10] Patavino T, Brady DM (2001). Natural medicine and nutritional therapy as an alternative treatment in systemic lupus erythematosus. Altern Med Rev.

